# *trans*-Bis[4-amino-*N*-(pyrimidin-2-yl)benzene­sulfonamidato]dipyridine­nickel(II) hemihydrate

**DOI:** 10.1107/S1600536809043621

**Published:** 2009-11-14

**Authors:** Yan-Fei Wang, Fu-Xing Li, Yan Peng, Zhen-Feng Chen, Hong Liang

**Affiliations:** a School of Chemistry and Chemical Engineering, Central South University, Changsha 410083, People’s Republic of China; b Key Laboratory for the Chemistry and Molecular Engineering of Medicinal Resources (Ministry of Education), School of Chemistry & Chemical Engineering, Guangxi Normal University, Guilin 541004, People’s Republic of China

## Abstract

The asymmetric unit of the title compound, [Ni(C_10_H_9_N_4_O_2_S)_2_(C_5_H_5_N)_2_]·0.5H_2_O, contains the distorted octa­hedral *trans*-[Ni(sdz)_2_(py)_2_] (sdz is the sulfadiazine anion and py is pyridine) complex mol­ecule and half of a water mol­ecule. A three-dimensional network is generated by N—H⋯O and O—H⋯O hydrogen bonds and C—H⋯O inter­actions between the complex and the water mol­ecules.

## Related literature

For a sulfamerazine–nickel(II) complex, see: Hossain & Amoroso (2006 ▶). For sulfadiazine–metal complexes, see: Ajibade *et al.* (2006 ▶); Hossain *et al.* (2006 ▶); Yuan *et al.* (2001 ▶).
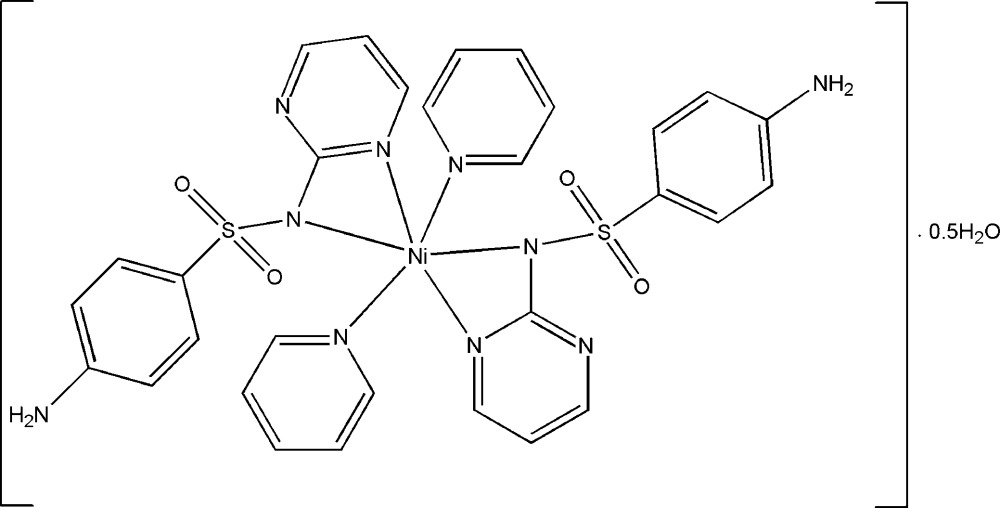


## Experimental

### 

#### Crystal data

[Ni(C_10_H_9_N_4_O_2_S)_2_(C_5_H_5_N)_2_]·0.5H_2_O
*M* *_r_* = 724.46
Monoclinic, 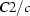

*a* = 39.593 (5) Å
*b* = 11.2297 (13) Å
*c* = 14.5656 (18) Å
β = 105.463 (2)°
*V* = 6241.6 (13) Å^3^
*Z* = 8
Mo *K*α radiation
μ = 0.81 mm^−1^
*T* = 193 K
0.39 × 0.30 × 0.30 mm

#### Data collection

Rigaku Mercury CCD diffractometer
Absorption correction: multi-scan (REQAB; Jacobson, 1998 ▶) *T*_min_ = 0.632, *T*_max_ = 0.784
29798 measured reflections
5701 independent reflections
5115 reflections with *I* > 2σ(*I*)
*R*_int_ = 0.045

#### Refinement

*R*[*F* ^2^ > 2σ(*F* ^2^)] = 0.043
*wR*(*F* ^2^) = 0.088
*S* = 1.15
5701 reflections
434 parameters
1 restraint
H atoms treated by a mixture of independent and constrained refinement
Δρ_max_ = 0.35 e Å^−3^
Δρ_min_ = −0.37 e Å^−3^

### 

Data collection: *CrystalClear* (Rigaku, 1999 ▶); cell refinement: *CrystalClear*; data reduction: *CrystalStructure* (Rigaku/MSC, 2000 ▶); program(s) used to solve structure: *SHELXS97* (Sheldrick, 2008 ▶); program(s) used to refine structure: *SHELXL97* (Sheldrick, 2008 ▶); molecular graphics: *SHELXTL* (Sheldrick, 2008 ▶); software used to prepare material for publication: *SHELXTL*.

## Supplementary Material

Crystal structure: contains datablocks I, global. DOI: 10.1107/S1600536809043621/pk2197sup1.cif

Structure factors: contains datablocks I. DOI: 10.1107/S1600536809043621/pk2197Isup2.hkl

Additional supplementary materials:  crystallographic information; 3D view; checkCIF report

## Figures and Tables

**Table 1:** Hydrogen-bond geometry (Å, °)

*D*—H⋯*A*	*D*—H	H⋯*A*	*D*⋯*A*	*D*—H⋯*A*
N8—H8*B*⋯O3^i^	0.88	2.28	3.091 (3)	153
N8—H8*A*⋯O4^ii^	0.88	2.44	3.287 (3)	161
N4—H4*B*⋯O5^iii^	0.88	2.26	3.113 (4)	162
N4—H4*A*⋯O1^iv^	0.88	2.54	3.075 (3)	120
O5—H5*A*⋯O2	0.83 (4)	1.97 (4)	2.791 (3)	172 (4)
C2—H2⋯O2^v^	0.95	2.45	3.280 (3)	145
C12—H12⋯O3^vi^	0.95	2.49	3.417 (3)	165

Symmetry codes: (i) 
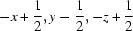
; (ii) 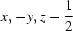; (iii) 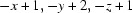; (iv) 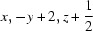; (v) 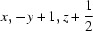; (vi) 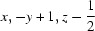.

**Fig. 1.**
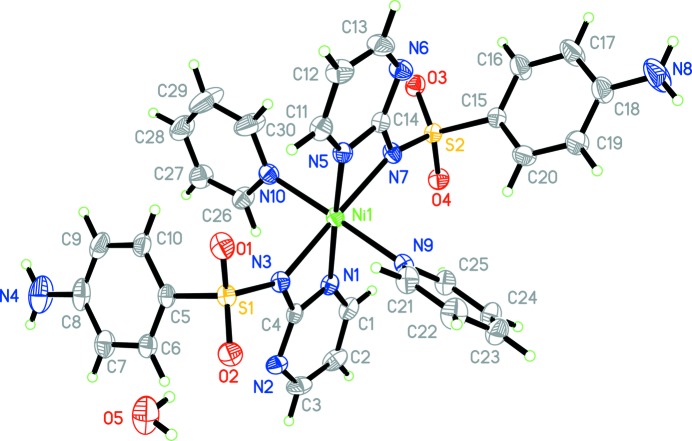
The molecular structure, showing the atom-labelling scheme. Displacement ellipsoids are drawn at the 50% probability level.

**Fig. 2.**
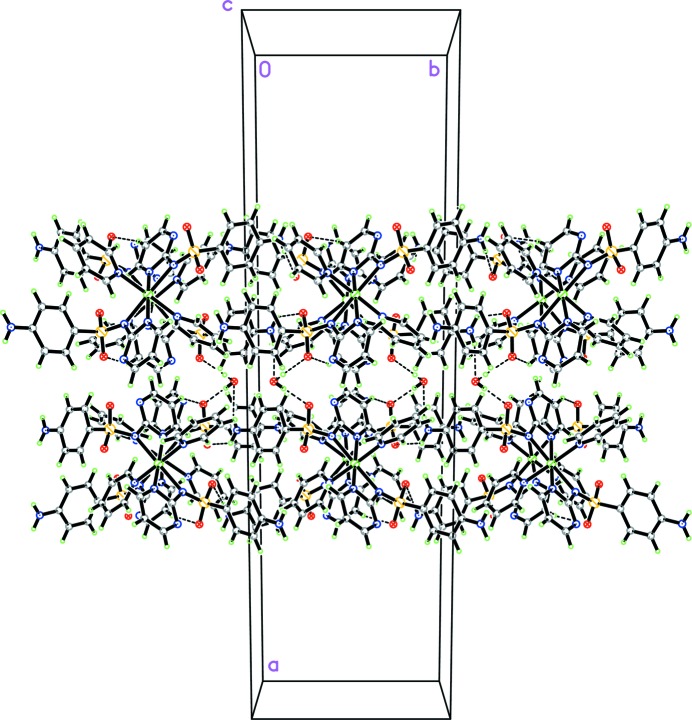
A packing plot of the title compound viewed along [001]. Hydrogen bonds are shown as dashed lines.

## supplementary crystallographic information

### Comment

The title compound consists of [Ni(C_11_H_11_N_4_O_4_S_2_] and 0.5 lattice water molecule. Similar to *trans*-[Ni(smr)_2_(py)_2_] (where smr = sulfamerazinate anion and py = pyridine) (Hossain & Amoroso, 2006), the title nickel(II) complex has a six-coordinated distorted octahedral geometry and contains two bidentate N-coordinated sulfadiazinate anions and two pyridine molecules occupying the *trans* sites. The coordination mode of sulfadiazine is similar to its cobalt(II) complex (Ajibade *et al.*, 2006), but different from Zn(sdz)_2_ (Yuan *et al.*, 2001) and its copper complex (Hossain *et al.* 2006). The Ni—N bond distances involving the sulfonamide atoms N3, N7,the pyrimido atoms N1, N5, and the pyridine atoms N9, N10, are very similar, at 2.083 (2), 2.122 (2), 2.109 (2), 2.070 (2), 2.134 (2), 2.159 (2) Å, respectively. The tetrahedral coordination at S is distorted, as in the neutral sulfadiazine molecule. A three dimensional network is generated *via* N—H···O and O—H···O hydrogen bonds and C—H···O interactions between the complex and water molecules.

### Experimental

0.2 mmol Ni(NO_3_)_2_.6H_2_O, 0.4 mmol sulfadiazine, ethanol (2 ml) and pyridine (0.2 ml) were placed in a Pyrex tube (*ca* 20 cm). The tube was frozen with liquid N_2_, evacuated under vacuum, sealed with a torch and heated at 353 K for three days to give light-blue block-shaped crystals, with a yield of 55%.

### Refinement

The water H atoms were found in a difference Fourier map and refined freely. Other H atoms were treated as riding, with C—H distances of 0.95 Å and N—H distances of 0.88 Å, and were refined using a riding model with *U*_iso_(H) = 1.2*U*_eq_ (C and N).

### Crystal data

**Table:**

[Ni(C_10_H_9_N_4_O_2_S)_2_(C_5_H_5_N)_2_]·0.5H_2_O	*F*(000) = 3000
*M**_r_* = 724.46	*D*_x_ = 1.542 Mg m^−^^3^
Monoclinic, *C*2/*c*	Mo *K*α radiation, λ = 0.71070 Å
Hall symbol: -C 2yc	Cell parameters from 10593 reflections
*a* = 39.593 (5) Å	θ = 3.0–25.3°
*b* = 11.2297 (13) Å	µ = 0.81 mm^−^^1^
*c* = 14.5656 (18) Å	*T* = 193 K
β = 105.463 (2)°	Block, light-blue
*V* = 6241.6 (13) Å^3^	0.39 × 0.30 × 0.30 mm
*Z* = 8	

### Data collection

**Table:**

Rigaku Mercury CCD diffractometer	5701 independent reflections
Radiation source: fine-focus sealed tube	5115 reflections with *I* > 2σ(*I*)
graphite	*R*_int_ = 0.045
Detector resolution: 7.31 pixels mm^-1^	θ_max_ = 25.4°, θ_min_ = 3.0°
ω scans	*h* = −47→46
Absorption correction: multi-scan (REQAB; Jacobson, 1998)	*k* = −13→13
*T*_min_ = 0.632, *T*_max_ = 0.784	*l* = −15→17
29798 measured reflections	

### Refinement

**Table:**

Refinement on *F*^2^	Primary atom site location: structure-invariant direct methods
Least-squares matrix: full	Secondary atom site location: difference Fourier map
*R*[*F*^2^ > 2σ(*F*^2^)] = 0.043	Hydrogen site location: inferred from neighbouring sites
*wR*(*F*^2^) = 0.088	H atoms treated by a mixture of independent and constrained refinement
*S* = 1.15	*w* = 1/[σ^2^(*F*_o_^2^) + (0.0251*P*)^2^ + 12.1467*P*] where *P* = (*F*_o_^2^ + 2*F*_c_^2^)/3
5701 reflections	(Δ/σ)_max_ = 0.001
434 parameters	Δρ_max_ = 0.35 e Å^−^^3^
1 restraint	Δρ_min_ = −0.37 e Å^−^^3^

### Special details

**Table:**

Geometry. All e.s.d.'s (except the e.s.d. in the dihedral angle between two l.s. planes) are estimated using the full covariance matrix. The cell e.s.d.'s are taken into account individually in the estimation of e.s.d.'s in distances, angles and torsion angles; correlations between e.s.d.'s in cell parameters are only used when they are defined by crystal symmetry. An approximate (isotropic) treatment of cell e.s.d.'s is used for estimating e.s.d.'s involving l.s. planes.
Refinement. Refinement of *F*^2^ against ALL reflections. The weighted *R*-factor *wR* and goodness of fit *S* are based on *F*^2^, conventional *R*-factors *R* are based on *F*, with *F* set to zero for negative *F*^2^. The threshold expression of *F*^2^ > σ(*F*^2^) is used only for calculating *R*-factors(gt) *etc*. and is not relevant to the choice of reflections for refinement. *R*-factors based on *F*^2^ are statistically about twice as large as those based on *F*, and *R*- factors based on ALL data will be even larger.

### Fractional atomic coordinates and isotropic or equivalent isotropic displacement parameters (Å^2^)

**Table:**

	*x*	*y*	*z*	*U*_iso_*/*U*_eq_	
Ni1	0.376495 (8)	0.47975 (3)	0.36660 (2)	0.02041 (10)	
S1	0.428806 (17)	0.71628 (6)	0.33576 (4)	0.02185 (16)	
S2	0.317022 (17)	0.25094 (6)	0.38453 (4)	0.02043 (15)	
O1	0.40113 (5)	0.73522 (17)	0.24982 (13)	0.0314 (5)	
O2	0.46403 (5)	0.71068 (17)	0.32506 (13)	0.0297 (5)	
O3	0.28333 (5)	0.28191 (16)	0.39866 (13)	0.0263 (4)	
O4	0.34354 (5)	0.21802 (16)	0.47017 (12)	0.0267 (4)	
O5	0.5000	0.8828 (3)	0.2500	0.0490 (9)	
H5A	0.4897 (10)	0.836 (3)	0.277 (3)	0.084 (16)*	
N1	0.41630 (6)	0.46846 (19)	0.49554 (15)	0.0217 (5)	
N2	0.46869 (5)	0.5851 (2)	0.51753 (15)	0.0249 (5)	
N3	0.41832 (5)	0.59728 (19)	0.38171 (14)	0.0212 (5)	
N4	0.42496 (9)	1.0955 (2)	0.6134 (2)	0.0584 (9)	
H4A	0.4050	1.1303	0.6132	0.070*	
H4B	0.4444	1.1170	0.6553	0.070*	
N5	0.34162 (5)	0.48874 (19)	0.23269 (15)	0.0221 (5)	
N6	0.28946 (6)	0.3704 (2)	0.18929 (16)	0.0268 (5)	
N7	0.33422 (6)	0.35699 (19)	0.33871 (15)	0.0226 (5)	
N8	0.29208 (8)	−0.1432 (3)	0.1044 (2)	0.0651 (10)	
H8A	0.3092	−0.1693	0.0818	0.078*	
H8B	0.2711	−0.1751	0.0850	0.078*	
N9	0.40712 (6)	0.34694 (19)	0.32133 (15)	0.0225 (5)	
N10	0.34701 (6)	0.6123 (2)	0.41977 (16)	0.0247 (5)	
C1	0.43038 (7)	0.4097 (2)	0.57599 (18)	0.0265 (6)	
H1	0.4170	0.3504	0.5970	0.032*	
C2	0.46398 (8)	0.4336 (3)	0.6293 (2)	0.0320 (7)	
H2	0.4744	0.3905	0.6858	0.038*	
C3	0.48177 (7)	0.5226 (3)	0.59712 (19)	0.0309 (7)	
H3	0.5049	0.5408	0.6339	0.037*	
C4	0.43644 (7)	0.5526 (2)	0.46787 (18)	0.0203 (6)	
C5	0.42814 (7)	0.8321 (2)	0.41556 (18)	0.0224 (6)	
C6	0.45869 (7)	0.8664 (2)	0.48213 (19)	0.0266 (6)	
H6	0.4804	0.8298	0.4825	0.032*	
C7	0.45746 (8)	0.9540 (3)	0.5479 (2)	0.0335 (7)	
H7	0.4784	0.9779	0.5932	0.040*	
C8	0.42570 (9)	1.0076 (3)	0.5483 (2)	0.0372 (8)	
C9	0.39532 (8)	0.9720 (3)	0.4813 (2)	0.0375 (8)	
H9	0.3736	1.0080	0.4811	0.045*	
C10	0.39631 (8)	0.8855 (2)	0.4155 (2)	0.0303 (7)	
H10	0.3754	0.8620	0.3701	0.036*	
C11	0.33173 (7)	0.5489 (2)	0.15111 (19)	0.0271 (6)	
H11	0.3462	0.6109	0.1385	0.033*	
C12	0.30078 (7)	0.5223 (3)	0.0849 (2)	0.0313 (7)	
H12	0.2934	0.5641	0.0263	0.038*	
C13	0.28092 (8)	0.4319 (3)	0.1077 (2)	0.0328 (7)	
H13	0.2596	0.4119	0.0623	0.039*	
C14	0.31993 (7)	0.4022 (2)	0.24983 (18)	0.0216 (6)	
C15	0.30996 (7)	0.1324 (2)	0.30324 (18)	0.0218 (6)	
C16	0.27727 (8)	0.0820 (3)	0.2716 (2)	0.0379 (8)	
H16	0.2586	0.1105	0.2950	0.046*	
C17	0.27131 (8)	−0.0095 (3)	0.2061 (3)	0.0466 (9)	
H17	0.2486	−0.0435	0.1853	0.056*	
C18	0.29810 (8)	−0.0527 (3)	0.1700 (2)	0.0350 (7)	
C19	0.33103 (8)	−0.0008 (3)	0.2019 (2)	0.0336 (7)	
H19	0.3498	−0.0283	0.1782	0.040*	
C20	0.33674 (7)	0.0903 (3)	0.2676 (2)	0.0310 (7)	
H20	0.3594	0.1247	0.2887	0.037*	
C21	0.41867 (7)	0.3623 (3)	0.24379 (19)	0.0289 (6)	
H21	0.4128	0.4340	0.2085	0.035*	
C22	0.43874 (8)	0.2791 (3)	0.2126 (2)	0.0374 (7)	
H22	0.4459	0.2926	0.1562	0.045*	
C23	0.44820 (8)	0.1763 (3)	0.2645 (2)	0.0330 (7)	
H23	0.4623	0.1182	0.2451	0.040*	
C24	0.43681 (8)	0.1593 (3)	0.3451 (2)	0.0306 (7)	
H24	0.4429	0.0893	0.3825	0.037*	
C25	0.41634 (7)	0.2460 (2)	0.37019 (19)	0.0268 (6)	
H25	0.4083	0.2333	0.4254	0.032*	
C26	0.35952 (8)	0.6565 (3)	0.5073 (2)	0.0316 (7)	
H26	0.3806	0.6234	0.5461	0.038*	
C27	0.34397 (8)	0.7466 (3)	0.5450 (2)	0.0362 (7)	
H27	0.3541	0.7746	0.6078	0.043*	
C28	0.31366 (8)	0.7953 (3)	0.4905 (2)	0.0437 (8)	
H28	0.3024	0.8584	0.5142	0.052*	
C29	0.29995 (10)	0.7508 (4)	0.4010 (3)	0.0622 (12)	
H29	0.2787	0.7820	0.3614	0.075*	
C30	0.31719 (8)	0.6605 (3)	0.3687 (2)	0.0504 (10)	
H30	0.3073	0.6308	0.3063	0.060*	

### Atomic displacement parameters (Å^2^)

**Table:**

	*U*^11^	*U*^22^	*U*^33^	*U*^12^	*U*^13^	*U*^23^
Ni1	0.02345 (19)	0.01857 (18)	0.01829 (18)	−0.00101 (14)	0.00399 (14)	0.00093 (14)
S1	0.0273 (4)	0.0209 (3)	0.0187 (3)	−0.0014 (3)	0.0084 (3)	0.0012 (3)
S2	0.0248 (3)	0.0191 (3)	0.0181 (3)	0.0005 (3)	0.0070 (3)	0.0016 (3)
O1	0.0407 (12)	0.0291 (11)	0.0211 (10)	−0.0029 (9)	0.0026 (9)	0.0065 (8)
O2	0.0299 (11)	0.0324 (11)	0.0329 (11)	−0.0021 (9)	0.0192 (9)	−0.0045 (9)
O3	0.0276 (10)	0.0256 (10)	0.0291 (10)	0.0025 (8)	0.0134 (9)	−0.0007 (8)
O4	0.0317 (10)	0.0270 (10)	0.0192 (10)	0.0024 (8)	0.0027 (8)	0.0043 (8)
O5	0.071 (3)	0.033 (2)	0.053 (2)	0.000	0.035 (2)	0.000
N1	0.0275 (12)	0.0201 (12)	0.0181 (11)	0.0036 (9)	0.0071 (10)	0.0026 (9)
N2	0.0216 (12)	0.0291 (13)	0.0231 (12)	0.0032 (10)	0.0042 (10)	−0.0008 (10)
N3	0.0257 (12)	0.0202 (12)	0.0162 (11)	0.0009 (9)	0.0030 (9)	0.0028 (9)
N4	0.090 (2)	0.0377 (17)	0.0558 (19)	0.0075 (16)	0.0342 (18)	−0.0174 (15)
N5	0.0237 (12)	0.0209 (12)	0.0219 (12)	0.0001 (9)	0.0064 (10)	0.0013 (9)
N6	0.0254 (12)	0.0299 (13)	0.0231 (12)	−0.0027 (10)	0.0031 (10)	0.0037 (10)
N7	0.0275 (12)	0.0194 (12)	0.0192 (11)	−0.0035 (9)	0.0032 (10)	0.0029 (9)
N8	0.0504 (18)	0.076 (2)	0.078 (2)	−0.0238 (17)	0.0320 (17)	−0.0562 (19)
N9	0.0258 (12)	0.0209 (12)	0.0202 (11)	−0.0040 (9)	0.0053 (10)	0.0000 (9)
N10	0.0249 (12)	0.0242 (12)	0.0254 (13)	−0.0023 (10)	0.0076 (10)	0.0009 (10)
C1	0.0355 (16)	0.0254 (15)	0.0208 (14)	0.0074 (12)	0.0110 (13)	0.0042 (12)
C2	0.0341 (16)	0.0401 (17)	0.0213 (15)	0.0132 (14)	0.0064 (13)	0.0082 (13)
C3	0.0223 (14)	0.0453 (18)	0.0225 (15)	0.0061 (13)	0.0016 (12)	−0.0034 (13)
C4	0.0257 (14)	0.0174 (13)	0.0189 (13)	0.0031 (11)	0.0079 (11)	−0.0020 (11)
C5	0.0318 (15)	0.0169 (13)	0.0208 (14)	−0.0008 (11)	0.0111 (12)	0.0026 (11)
C6	0.0329 (16)	0.0216 (14)	0.0267 (15)	0.0010 (12)	0.0103 (13)	0.0024 (12)
C7	0.0480 (19)	0.0260 (16)	0.0256 (15)	0.0008 (14)	0.0082 (14)	−0.0013 (13)
C8	0.065 (2)	0.0203 (15)	0.0359 (17)	0.0015 (15)	0.0303 (17)	0.0018 (13)
C9	0.0407 (18)	0.0240 (16)	0.057 (2)	0.0090 (14)	0.0297 (17)	0.0065 (15)
C10	0.0314 (16)	0.0193 (14)	0.0429 (18)	0.0021 (12)	0.0146 (14)	0.0045 (13)
C11	0.0325 (16)	0.0257 (15)	0.0247 (15)	0.0014 (12)	0.0101 (13)	0.0058 (12)
C12	0.0333 (16)	0.0382 (17)	0.0207 (14)	0.0028 (14)	0.0042 (13)	0.0115 (13)
C13	0.0283 (15)	0.0400 (18)	0.0254 (15)	−0.0014 (14)	−0.0013 (13)	0.0026 (14)
C14	0.0242 (14)	0.0195 (14)	0.0215 (14)	0.0010 (11)	0.0070 (11)	0.0001 (11)
C15	0.0254 (14)	0.0218 (14)	0.0181 (13)	0.0005 (11)	0.0056 (11)	0.0003 (11)
C16	0.0317 (16)	0.0361 (18)	0.053 (2)	−0.0107 (14)	0.0232 (15)	−0.0177 (15)
C17	0.0335 (17)	0.052 (2)	0.061 (2)	−0.0178 (16)	0.0235 (17)	−0.0289 (18)
C18	0.0387 (17)	0.0328 (17)	0.0348 (17)	−0.0049 (14)	0.0122 (14)	−0.0100 (14)
C19	0.0313 (16)	0.0405 (18)	0.0322 (16)	0.0001 (14)	0.0140 (13)	−0.0129 (14)
C20	0.0231 (14)	0.0376 (17)	0.0321 (16)	−0.0033 (13)	0.0070 (13)	−0.0079 (14)
C21	0.0405 (17)	0.0268 (15)	0.0192 (14)	0.0010 (13)	0.0076 (13)	0.0043 (12)
C22	0.0501 (19)	0.0391 (18)	0.0300 (17)	0.0010 (15)	0.0226 (15)	0.0003 (14)
C23	0.0369 (17)	0.0302 (16)	0.0352 (17)	0.0046 (13)	0.0151 (14)	−0.0029 (14)
C24	0.0389 (17)	0.0239 (15)	0.0295 (16)	0.0050 (13)	0.0098 (13)	0.0036 (12)
C25	0.0360 (16)	0.0243 (15)	0.0228 (14)	−0.0005 (12)	0.0124 (13)	0.0033 (12)
C26	0.0328 (16)	0.0324 (17)	0.0293 (16)	0.0060 (13)	0.0080 (13)	−0.0005 (13)
C27	0.0377 (17)	0.0398 (18)	0.0323 (17)	0.0043 (15)	0.0115 (14)	−0.0072 (14)
C28	0.0415 (19)	0.042 (2)	0.049 (2)	0.0133 (16)	0.0139 (16)	−0.0086 (16)
C29	0.052 (2)	0.078 (3)	0.046 (2)	0.041 (2)	−0.0041 (18)	−0.016 (2)
C30	0.0371 (19)	0.065 (2)	0.041 (2)	0.0211 (17)	−0.0049 (16)	−0.0147 (18)

### Geometric parameters (Å, °)

**Table:**

Ni1—N5	2.070 (2)	C6—C7	1.384 (4)
Ni1—N3	2.083 (2)	C6—H6	0.9500
Ni1—N1	2.109 (2)	C7—C8	1.395 (4)
Ni1—N7	2.122 (2)	C7—H7	0.9500
Ni1—N9	2.134 (2)	C8—C9	1.390 (5)
Ni1—N10	2.159 (2)	C9—C10	1.372 (4)
S1—O1	1.443 (2)	C9—H9	0.9500
S1—O2	1.4454 (19)	C10—H10	0.9500
S1—N3	1.598 (2)	C11—C12	1.375 (4)
S1—C5	1.749 (3)	C11—H11	0.9500
S2—O3	1.4455 (19)	C12—C13	1.377 (4)
S2—O4	1.4480 (19)	C12—H12	0.9500
S2—N7	1.603 (2)	C13—H13	0.9500
S2—C15	1.754 (3)	C15—C16	1.375 (4)
O5—H5A	0.83 (4)	C15—C20	1.383 (4)
N1—C1	1.331 (3)	C16—C17	1.379 (4)
N1—C4	1.365 (3)	C16—H16	0.9500
N2—C3	1.336 (3)	C17—C18	1.391 (4)
N2—C4	1.339 (3)	C17—H17	0.9500
N3—C4	1.364 (3)	C18—C19	1.390 (4)
N4—C8	1.374 (4)	C19—C20	1.377 (4)
N4—H4A	0.8800	C19—H19	0.9500
N4—H4B	0.8800	C20—H20	0.9500
N5—C11	1.332 (3)	C21—C22	1.380 (4)
N5—C14	1.363 (3)	C21—H21	0.9500
N6—C13	1.337 (4)	C22—C23	1.376 (4)
N6—C14	1.339 (3)	C22—H22	0.9500
N7—C14	1.365 (3)	C23—C24	1.379 (4)
N8—C18	1.371 (4)	C23—H23	0.9500
N8—H8A	0.8800	C24—C25	1.378 (4)
N8—H8B	0.8800	C24—H24	0.9500
N9—C25	1.337 (3)	C25—H25	0.9500
N9—C21	1.338 (3)	C26—C27	1.373 (4)
N10—C30	1.331 (4)	C26—H26	0.9500
N10—C26	1.334 (4)	C27—C28	1.364 (4)
C1—C2	1.376 (4)	C27—H27	0.9500
C1—H1	0.9500	C28—C29	1.367 (5)
C2—C3	1.375 (4)	C28—H28	0.9500
C2—H2	0.9500	C29—C30	1.373 (5)
C3—H3	0.9500	C29—H29	0.9500
C5—C6	1.388 (4)	C30—H30	0.9500
C5—C10	1.396 (4)		
			
N5—Ni1—N3	112.36 (8)	C6—C7—H7	119.7
N5—Ni1—N1	173.86 (8)	C8—C7—H7	119.7
N3—Ni1—N1	63.88 (8)	N4—C8—C9	121.2 (3)
N5—Ni1—N7	63.73 (8)	N4—C8—C7	119.8 (3)
N3—Ni1—N7	174.89 (8)	C9—C8—C7	119.0 (3)
N1—Ni1—N7	119.69 (8)	C10—C9—C8	120.9 (3)
N5—Ni1—N9	91.96 (8)	C10—C9—H9	119.5
N3—Ni1—N9	88.53 (8)	C8—C9—H9	119.5
N1—Ni1—N9	83.22 (8)	C9—C10—C5	119.8 (3)
N7—Ni1—N9	88.34 (8)	C9—C10—H10	120.1
N5—Ni1—N10	90.90 (8)	C5—C10—H10	120.1
N3—Ni1—N10	90.92 (8)	N5—C11—C12	120.7 (3)
N1—Ni1—N10	93.96 (8)	N5—C11—H11	119.7
N7—Ni1—N10	92.41 (8)	C12—C11—H11	119.7
N9—Ni1—N10	177.09 (8)	C11—C12—C13	116.8 (3)
O1—S1—O2	116.38 (12)	C11—C12—H12	121.6
O1—S1—N3	105.35 (11)	C13—C12—H12	121.6
O2—S1—N3	112.00 (12)	N6—C13—C12	124.7 (3)
O1—S1—C5	108.93 (12)	N6—C13—H13	117.6
O2—S1—C5	106.85 (12)	C12—C13—H13	117.6
N3—S1—C5	106.97 (11)	N6—C14—N5	124.8 (2)
O3—S2—O4	115.06 (11)	N6—C14—N7	126.7 (2)
O3—S2—N7	113.02 (11)	N5—C14—N7	108.5 (2)
O4—S2—N7	104.92 (11)	C16—C15—C20	118.8 (3)
O3—S2—C15	106.95 (12)	C16—C15—S2	120.4 (2)
O4—S2—C15	109.65 (12)	C20—C15—S2	120.9 (2)
N7—S2—C15	106.97 (12)	C15—C16—C17	120.7 (3)
C1—N1—C4	117.6 (2)	C15—C16—H16	119.6
C1—N1—Ni1	148.4 (2)	C17—C16—H16	119.6
C4—N1—Ni1	92.99 (15)	C16—C17—C18	121.0 (3)
C3—N2—C4	114.8 (2)	C16—C17—H17	119.5
C4—N3—S1	124.13 (18)	C18—C17—H17	119.5
C4—N3—Ni1	94.16 (15)	N8—C18—C19	121.1 (3)
S1—N3—Ni1	140.67 (12)	N8—C18—C17	120.9 (3)
C8—N4—H4A	120.0	C19—C18—C17	118.0 (3)
C8—N4—H4B	120.0	C20—C19—C18	120.6 (3)
H4A—N4—H4B	120.0	C20—C19—H19	119.7
C11—N5—C14	118.4 (2)	C18—C19—H19	119.7
C11—N5—Ni1	146.01 (19)	C19—C20—C15	121.0 (3)
C14—N5—Ni1	95.00 (15)	C19—C20—H20	119.5
C13—N6—C14	114.7 (2)	C15—C20—H20	119.5
C14—N7—S2	123.83 (18)	N9—C21—C22	123.2 (3)
C14—N7—Ni1	92.64 (15)	N9—C21—H21	118.4
S2—N7—Ni1	143.48 (13)	C22—C21—H21	118.4
C18—N8—H8A	120.0	C23—C22—C21	118.9 (3)
C18—N8—H8B	120.0	C23—C22—H22	120.5
H8A—N8—H8B	120.0	C21—C22—H22	120.5
C25—N9—C21	116.9 (2)	C22—C23—C24	118.8 (3)
C25—N9—Ni1	121.68 (18)	C22—C23—H23	120.6
C21—N9—Ni1	121.38 (18)	C24—C23—H23	120.6
C30—N10—C26	115.5 (3)	C25—C24—C23	118.5 (3)
C30—N10—Ni1	124.3 (2)	C25—C24—H24	120.7
C26—N10—Ni1	120.04 (18)	C23—C24—H24	120.7
N1—C1—C2	121.0 (3)	N9—C25—C24	123.7 (3)
N1—C1—H1	119.5	N9—C25—H25	118.2
C2—C1—H1	119.5	C24—C25—H25	118.2
C3—C2—C1	117.1 (3)	N10—C26—C27	124.4 (3)
C3—C2—H2	121.4	N10—C26—H26	117.8
C1—C2—H2	121.4	C27—C26—H26	117.8
N2—C3—C2	124.2 (3)	C28—C27—C26	118.8 (3)
N2—C3—H3	117.9	C28—C27—H27	120.6
C2—C3—H3	117.9	C26—C27—H27	120.6
N2—C4—N3	126.1 (2)	C27—C28—C29	118.2 (3)
N2—C4—N1	125.2 (2)	C27—C28—H28	120.9
N3—C4—N1	108.7 (2)	C29—C28—H28	120.9
C6—C5—C10	120.0 (3)	C28—C29—C30	119.3 (3)
C6—C5—S1	120.4 (2)	C28—C29—H29	120.4
C10—C5—S1	119.5 (2)	C30—C29—H29	120.4
C7—C6—C5	119.7 (3)	N10—C30—C29	123.9 (3)
C7—C6—H6	120.1	N10—C30—H30	118.1
C5—C6—H6	120.1	C29—C30—H30	118.1
C6—C7—C8	120.5 (3)		
			
N3—Ni1—N1—C1	−169.1 (4)	S1—N3—C4—N1	165.80 (17)
N7—Ni1—N1—C1	6.7 (4)	Ni1—N3—C4—N1	−4.7 (2)
N9—Ni1—N1—C1	−77.5 (3)	C1—N1—C4—N2	−3.4 (4)
N10—Ni1—N1—C1	101.8 (3)	Ni1—N1—C4—N2	−175.2 (2)
N3—Ni1—N1—C4	−3.18 (14)	C1—N1—C4—N3	176.4 (2)
N7—Ni1—N1—C4	172.69 (14)	Ni1—N1—C4—N3	4.6 (2)
N9—Ni1—N1—C4	88.51 (15)	O1—S1—C5—C6	−151.9 (2)
N10—Ni1—N1—C4	−92.26 (15)	O2—S1—C5—C6	−25.5 (2)
O1—S1—N3—C4	−175.1 (2)	N3—S1—C5—C6	94.6 (2)
O2—S1—N3—C4	57.5 (2)	O1—S1—C5—C10	31.7 (2)
C5—S1—N3—C4	−59.3 (2)	O2—S1—C5—C10	158.2 (2)
O1—S1—N3—Ni1	−10.2 (2)	N3—S1—C5—C10	−81.7 (2)
O2—S1—N3—Ni1	−137.63 (18)	C10—C5—C6—C7	−0.4 (4)
C5—S1—N3—Ni1	105.6 (2)	S1—C5—C6—C7	−176.7 (2)
N5—Ni1—N3—C4	−171.50 (14)	C5—C6—C7—C8	0.5 (4)
N1—Ni1—N3—C4	3.18 (14)	C6—C7—C8—N4	−179.3 (3)
N9—Ni1—N3—C4	−79.99 (15)	C6—C7—C8—C9	−0.3 (4)
N10—Ni1—N3—C4	97.15 (15)	N4—C8—C9—C10	179.0 (3)
N5—Ni1—N3—S1	21.0 (2)	C7—C8—C9—C10	0.0 (4)
N1—Ni1—N3—S1	−164.3 (2)	C8—C9—C10—C5	0.1 (4)
N9—Ni1—N3—S1	112.5 (2)	C6—C5—C10—C9	0.1 (4)
N10—Ni1—N3—S1	−70.4 (2)	S1—C5—C10—C9	176.5 (2)
N3—Ni1—N5—C11	−11.8 (4)	C14—N5—C11—C12	−1.6 (4)
N7—Ni1—N5—C11	171.8 (4)	Ni1—N5—C11—C12	−169.5 (2)
N9—Ni1—N5—C11	−101.0 (3)	N5—C11—C12—C13	0.4 (4)
N10—Ni1—N5—C11	79.6 (3)	C14—N6—C13—C12	−0.7 (4)
N3—Ni1—N5—C14	178.81 (14)	C11—C12—C13—N6	0.8 (5)
N7—Ni1—N5—C14	2.42 (14)	C13—N6—C14—N5	−0.7 (4)
N9—Ni1—N5—C14	89.60 (15)	C13—N6—C14—N7	177.7 (3)
N10—Ni1—N5—C14	−89.82 (16)	C11—N5—C14—N6	1.8 (4)
O3—S2—N7—C14	−63.8 (2)	Ni1—N5—C14—N6	175.1 (2)
O4—S2—N7—C14	170.0 (2)	C11—N5—C14—N7	−176.8 (2)
C15—S2—N7—C14	53.6 (2)	Ni1—N5—C14—N7	−3.6 (2)
O3—S2—N7—Ni1	112.9 (2)	S2—N7—C14—N6	2.9 (4)
O4—S2—N7—Ni1	−13.2 (2)	Ni1—N7—C14—N6	−175.1 (2)
C15—S2—N7—Ni1	−129.7 (2)	S2—N7—C14—N5	−178.49 (18)
N5—Ni1—N7—C14	−2.41 (14)	Ni1—N7—C14—N5	3.5 (2)
N1—Ni1—N7—C14	−176.65 (14)	O3—S2—C15—C16	−4.6 (3)
N9—Ni1—N7—C14	−95.42 (15)	O4—S2—C15—C16	120.8 (2)
N10—Ni1—N7—C14	87.40 (15)	N7—S2—C15—C16	−126.0 (2)
N5—Ni1—N7—S2	−179.7 (3)	O3—S2—C15—C20	173.3 (2)
N1—Ni1—N7—S2	6.1 (3)	O4—S2—C15—C20	−61.3 (3)
N9—Ni1—N7—S2	87.3 (2)	N7—S2—C15—C20	52.0 (3)
N10—Ni1—N7—S2	−89.9 (2)	C20—C15—C16—C17	0.6 (5)
N5—Ni1—N9—C25	−129.8 (2)	S2—C15—C16—C17	178.6 (3)
N3—Ni1—N9—C25	117.9 (2)	C15—C16—C17—C18	−0.4 (5)
N1—Ni1—N9—C25	54.0 (2)	C16—C17—C18—N8	−179.6 (3)
N7—Ni1—N9—C25	−66.1 (2)	C16—C17—C18—C19	0.0 (5)
N5—Ni1—N9—C21	51.8 (2)	N8—C18—C19—C20	179.8 (3)
N3—Ni1—N9—C21	−60.5 (2)	C17—C18—C19—C20	0.3 (5)
N1—Ni1—N9—C21	−124.4 (2)	C18—C19—C20—C15	−0.1 (5)
N7—Ni1—N9—C21	115.5 (2)	C16—C15—C20—C19	−0.3 (4)
N5—Ni1—N10—C30	3.0 (3)	S2—C15—C20—C19	−178.3 (2)
N3—Ni1—N10—C30	115.4 (3)	C25—N9—C21—C22	1.0 (4)
N1—Ni1—N10—C30	179.2 (3)	Ni1—N9—C21—C22	179.5 (2)
N7—Ni1—N10—C30	−60.8 (3)	N9—C21—C22—C23	−1.6 (5)
N5—Ni1—N10—C26	−172.8 (2)	C21—C22—C23—C24	1.0 (5)
N3—Ni1—N10—C26	−60.4 (2)	C22—C23—C24—C25	0.1 (4)
N1—Ni1—N10—C26	3.4 (2)	C21—N9—C25—C24	0.2 (4)
N7—Ni1—N10—C26	123.4 (2)	Ni1—N9—C25—C24	−178.3 (2)
C4—N1—C1—C2	0.2 (4)	C23—C24—C25—N9	−0.8 (4)
Ni1—N1—C1—C2	164.4 (3)	C30—N10—C26—C27	−0.9 (4)
N1—C1—C2—C3	1.8 (4)	Ni1—N10—C26—C27	175.3 (2)
C4—N2—C3—C2	−1.7 (4)	N10—C26—C27—C28	0.1 (5)
C1—C2—C3—N2	−1.0 (4)	C26—C27—C28—C29	0.7 (5)
C3—N2—C4—N3	−175.7 (2)	C27—C28—C29—C30	−0.7 (6)
C3—N2—C4—N1	4.1 (4)	C26—N10—C30—C29	0.8 (5)
S1—N3—C4—N2	−14.4 (4)	Ni1—N10—C30—C29	−175.2 (3)
Ni1—N3—C4—N2	175.1 (2)	C28—C29—C30—N10	0.0 (7)

### Hydrogen-bond geometry (Å, °)

**Table:**

*D*—H···*A*	*D*—H	H···*A*	*D*···*A*	*D*—H···*A*
N8—H8B···O3^i^	0.88	2.28	3.091 (3)	153
N8—H8A···O4^ii^	0.88	2.44	3.287 (3)	161
N4—H4B···O5^iii^	0.88	2.26	3.113 (4)	162
N4—H4A···O1^iv^	0.88	2.54	3.075 (3)	120
O5—H5A···O2	0.83 (4)	1.97 (4)	2.791 (3)	172 (4)
C2—H2···O2^v^	0.95	2.45	3.280 (3)	145
C12—H12···O3^vi^	0.95	2.49	3.417 (3)	165

Symmetry codes: (i) −*x*+1/2, *y*−1/2, −*z*+1/2; (ii) *x*, −*y*, *z*−1/2; (iii) −*x*+1, −*y*+2, −*z*+1; (iv) *x*, −*y*+2, *z*+1/2; (v) *x*, −*y*+1, *z*+1/2; (vi) *x*, −*y*+1, *z*−1/2.
